# Profile of neurological disorders in a tertiary center of education in orthopedics

**DOI:** 10.1055/s-0042-1759763

**Published:** 2023-03-14

**Authors:** Celmir de Oliveira Vilaça, Fabio de Souza, Kelly Biancardini Gomes Barbato

**Affiliations:** 1 Universidade Federal do Rio de Janeiro, Serviço de Neurologia, Rio de Janeiro RJ, Brazil.; 2 Universidade Federal Fluminense, Programa de Pós-Graduação em Neurologia/Neurociências, Rio de Janeiro RJ, Brazil.; 3 Instituto de Traumatologia e Ortopedia Jammil Haddad, Rio de Janeiro RJ, Brazil.; 4 Universidade Federal do Estado do Rio de Janeiro, Rio de Janeiro RJ, Brazil.; 5 Escola Médica Souza Marques, Rio de Janeiro RJ, Brazil.

**Keywords:** Neurology, Orthopedics, Education, Medical, Peripheral Nervous System Diseases, Epilepsy, Neurologia, Ortopedia, Educação Médica, Doenças do Sistema Nervoso Periférico, Epilepsia

## Abstract

**Background** Neurological conditions can cause secondary orthopedic disorders and can result from orthopedic surgical procedures. In addition, misdiagnosis and overtreatment involve both specialties. Epidemiological studies of neurological patients in tertiary units are often performed in emergency departments of general hospitals or rehabilitation centers.

**Objective** Describe the clinical and epidemiologic profile of neurological disorders in a Brazilian federal tertiary center and education hospital in orthopedics in Rio de Janeiro.

**Methods** We performed a retrospective study of the medical records of patients attended by neurology specialists of the internal medicine's department of the National Institute of Traumatology and Orthopedics from February 2014 to March 2020.

**Results** We reviewed neurological referrals in the medical records of 1,349 patients in the period. The mean age of patients was 49.67 years (standard deviation [SD] ± 18.99). There was a predominance of females, corresponding to 751 (55.7%) patients. Regarding ethnicity, 684 (50.7%) participants were white, 550 (40.8%) non-white, and 115 (8.5%) non-classified. Peripheral neuropathies (34.1%), osteoarticular diseases (10%), epilepsy (8.3%), developmental disorders (7.9%), and neuromuscular diseases (7.3%) were the 5 groups with the largest numbers of cases.

**Conclusion** The sample consisted mostly of females and white individuals, and approximately one third of the cases were of peripheral neuropathies. Epidemiological studies in neurology from tertiary centers of another medical specialty can improve the professional development of both specialties. This interdisciplinary approach can also optimize resources, help avoid misdiagnosis, and reduce disability.

## Resumo

**Antecedentes** Condições neurológicas tanto podem causar distúrbios ortopédicos secundários como podem ser consequências de procedimentos cirúrgicos ortopédicos. Além disso, erros de diagnóstico e sobre tratamento também envolvem ambas as especialidades. Estudos epidemiológicos de atendimento neurológico em unidades terciárias de saúde são geralmente realizados em serviços de emergência ou em centros de reabilitação.

**Objetivo** Descrever o perfil clínico e epidemiológico de diagnósticos em neurologia em um centro terciário de saúde no Brasil e hospital de educação em ortopedia no Rio de Janeiro.

**Métodos** Realizamos um estudo retrospectivo com revisão de prontuários dos pacientes atendidos pela neurologia do setor de clínica médica do Instituto Nacional de Traumatologia e Ortopedia no período de fevereiro de 2014 a março de 2020.

**Resultados** Revisamos os prontuários de 1.349 pacientes atendidos pela neurologia no período. A média de idade dos pacientes foi de 49,67 anos (desvio padrão [DP] ± 18,99). Houve predomínio do sexo feminino correspondendo a 751 (55,7%) dos atendimentos. Quanto à etnia, a amostra foi composta de 684 (50,7%) de brancos, 550 (40,8%) de não brancos e 115 (8,5%) de não classificados. Neuropatias periféricas (34,1%), doenças osteoarticulares (10%), epilepsias (8,3%), transtornos do desenvolvimento (7,9%) e doenças neuromusculares (7,3%) corresponderam aos 5 grupos com os maiores números de casos.

**Conclusão** A amostra se constituiu predominante de indivíduos do sexo feminino, brancos, e cerca de um terço dos casos corresponderam às neuropatias periféricas. Estudos de perfil de atendimento neurológico em hospitais terciários de outra especialidade médica podem aperfeiçoar a capacitação de ambos os profissionais. Esta abordagem interdisciplinar também pode otimizar recursos, contribuir para evitar erros diagnósticos e reduzir incapacidades.

## INTRODUCTION

Many neurological diseases can lead to orthopedic complications, such as fractures resulting from traumas secondary to epilepsy or in cases of neuromuscular scoliosis (NMS) in individuals with cerebral palsy (CP). 1 2 Also, many neurological conditions are confused with orthopedic diseases. For example, clinical signs and symptoms of amyotrophic lateral sclerosis (ALS) or HTLV-associated myelopathy/tropical spastic paraparesis (HAM/TSP) can be misdiagnosed as cervical spondylotic myelopathy (CSM). 3 Neurological conditions may require orthopedic surgical treatment, for instance, carpal tunnel syndrome (CTS) or correction of neurological cavus foot in individuals with Charcot-Marie-Tooth (CMT) disease. 4 5 Orthopedic surgeries can also cause neurological complications such as foot drop resulting from peroneal nerve damage in knee surgeries 6 or low-output ischemic stroke due to such major orthopedic surgeries as hip or multi-level spine surgeries. 7 8

Epidemiological studies of neurological patients in tertiary units are often performed in emergency departments of general hospitals or rehabilitation centers. In general tertiary hospitals, headaches are the principal cause of referrals. 9 In rehabilitation centers, traumatic brain injuries from falls and motor vehicle accidents are the most frequent causes of referral for neurological care. 10 Studies of neurological profiles of diagnoses in tertiary hospitals of another medical subspecialty are rare. 9 10 11 Moreover, neurology education is part of the medical residency program in orthopedics in Canada and Brazil. 12 13

The objective of this study was to describe the profile of neurological disorders in a tertiary center of education in orthopedics of the Brazilian Unified Health System (SUS, in the Portuguese acronym) in the metropolitan region of Rio de Janeiro.

## METHODS

We reviewed the neurological referrals in the medical records of 1,349 patients at a tertiary SUS hospital specialized in orthopedic care (Instituto Nacional de Traumatologia e Ortopedia Jamil Haddad). We included outpatients and ward patients of all ages attended from February 2014 to March 2020. We excluded neurological visits to the intensive care unit (ICU). For statistical analysis, besides using sociodemographic data, such as age, gender, ethnicity, and education level (years of schooling), we divided the clinical diagnoses into 13 distinct groups after neurological evaluation: 1) cerebrovascular diseases, 2) epilepsy, 3) headaches, 4) neurocognitive and aging diseases, 5) neuromuscular diseases, 6) peripheral neuropathies, 7) movement disorders, 8) tumors of the nervous system, 9) demyelinating diseases, 10) neuroinfections, 11) developmental disorders, 12) osteoarticular diseases, and 13) psychiatric disorders. For individuals with more than one neurological diagnosis, we counted each one separately.

We performed statistical analyses of the sociodemographic and clinical characteristics obtained with the statistical package IBM SPSS Statistics for Windows, version 20.0 (IBM Corp., Armonk, NY, USA) and Microsoft Excel version 2010 (Microsoft Corporation, Redmond, WA, USA) software. We describe the results in frequencies, percentages, means, and standard deviations (SDs). The National Institute of Traumatology and Orthopedics ethics committee approved the work (CAAE number:39244820.4.0000.5273).

## RESULTS

The sample consisted of 1,389 individuals, of which 1,205 were outpatients and 144 were ward consultations. As a result, we obtained 1,665 diagnoses, divided among the 13 diagnostic groups. The patients' mean age was 49.67 years (SD ± 18.99), with a minimum of 8 months and a maximum of 96 years. The gender distribution was 751 (55.7%) female and 598 (44.3%) male patients. However, when we considered only the ward visits (144), there was a predominance of males (75; 52.08%) compared with females (69; 47.92%). Table 1 shows all the principal sociodemographic results.

**Table 1:** Sociodemographic data

Age	YEAR ± SD*
Minimum	0.66 year (8 months)
Maximum	96 years
Mean	49.67 years ± 18.99
**Gender by place of consultation**	**N (%)**
*Outpatients*	
Female	682 (50.6%)
Male	523 (38.7%)
*Ward patients*	
Female	69 (5.1%)
Male	75 (5.6%)
**Ethnicity**	**N (%)**
White	684 (50.7%)
Non-white	550 (40.8%)
Non-classified	115 (8.5%)
**Years of study**	**N (%)**
0–6	371 (27.5%)
7–10	443 (32.8%)
11–15	468 (34.7%)
Above 15	60 (4.4%)
Unknown	7 (0.5%)

Abbreviations: %, percentage; N, number of cases; SD, standard deviation.

Table 2 reveals the sample distribution by frequency and percentage of the clinical diagnoses in decreasing order and categorized into the study's 13 groups. Peripheral neuropathies accounted for little more than one third of the neurological referrals in this specialized orthopedic national reference center (34.1%). All other diagnoses were distributed among the other 12 groups, with a frequency equal to or less than 10% each.

**Table 2:** Cases distribution by groups of pathologies

	Number of cases	Percentage of cases
Peripheral neuropathies	568	34.1
Osteoarticular diseases	167	10.0
Epilepsies	138	8.3
Developmental disorders	132	7.9
Neuromuscular diseases	122	7.3
Cerebrovascular diseases	110	6.6
Movement disorders	101	6.1
Headaches	101	6.1
Psychiatric disorders	100	6.0
Neurocognitive and aging diseases	62	3.7
Neuroinfections	37	2.2
Tumors of the nervous system	21	1.3
Demyelinating diseases	6	0.4
Total cases	1,665*	100

Note: *Cases of two or more neurologic diagnoses in the same patient counted separately.

Table 3 shows the five groups of pathologies with the largest numbers of cases and the percentages of the three etiologies most commonly found in each group. In the peripheral neuropathies group, other common diagnoses not shown in Table 3 were diabetic neuropathy (74 cases), alcoholic neuropathy (29 cases), CMT (22 cases), peroneal neuropathy (22 cases), and traumatic brachial plexopathy (21 cases).

**Table 3:** The most frequent etiologies in the five groups with the largest number of diagnoses

	Cases	Percentage
**Peripheral neuropathies**	**N = 568**	**34.10%**
Carpal tunnel syndrome	103	18.10%
Idiopathic neuropathy	97	17.10%
Lumbar radiculopathy	89	15.70%
Others	279	49.10%
**Osteoarticular diseases**	**N = 167**	**10.00%**
Non-neurological cavus foot	40	24.00%
Lumbar spondylosis	34	20.40%
Cervical spondylosis	25	15.00%
Others	68	40.60%
**Epilepsies**	**N = 138**	**8.30%**
Primary epilepsy or developmental disorders	79	57.20%
Epilepsy from toxic-metabolic diseases	23	16.70%
Epilepsy from traumatic brain injuries	20	14.50%
Others	16	11.60%
**Developmental disorders**	**N = 132**	**7.90%**
Cerebral palsy	76	57.60%
Mental retardation	19	14.40%
Hydrocephalus	7	5.30%
Others	30	22.70%
**Neuromuscular diseases**	**N = 122**	**7.30%**
Cervical spondylotic myelopathy	43	35.20%
Neuromuscular scoliosis	15	12.30%
Amyotrophic lateral sclerosis	11	9.00%
Others	53	43.50%

To understand the age distribution in each group of pathologies, we illustrated it among the three etiologies most commonly found in the five groups with the largest number of cases ( Table 4 ).

**Table 4:** Age distribution of the three most commonly etiologies in the five groups with the largest number of diagnoses

		Cases by age		
		< 18 years old	> 18–60 years old	> 60 years old
**Peripheral neuropathies**	Carpal tunnel syndrome	0	61	42
	Idiopathic neuropathy	5	50	42
	Lumbar radiculopathy	4	65	20
**Osteoarticular diseases**	Non-neurological cavus foot	8	9	23
	Lumbar spondylosis	0	17	17
	Cervical spondylosis	0	15	10
**Epilepsies**	Primary epilepsy or developmental disorders	13	59	7
	Epilepsy from toxic-metabolic diseases	1	17	5
	Epilepsy from traumatic brain injuries	0	19	1
**Developmental disorders**	Cerebral palsy	46	29	1
	Mental retardation	7	12	0
	Hydrocephalus	3	4	0
**Neuromuscular diseases**	Cervical spondylotic myelopathy	0	23	20
	Neuromuscular scoliosis	5	10	0
	Amyotrophic lateral sclerosis	0	5	6

## DISCUSSION

Our sample was comprised mostly of females and white adults. Peripheral neuropathy was the most frequent diagnosis in this tertiary orthopedic education service.

Despite the predominance of females in the sample, there was a discrete male predominance when we considered only the ward visits. The higher risk of trauma in males, when compared to females, could explain this distribution difference with consequent urgent orthopedic hospitalization by fractures. 14 Although not shown in the sample results, there were 14 cases in the ward due to multiple trauma from motorcycle accidents, 11 male and 3 female patients. This risk may be associated with cargo activities or the direction of motor vehicles, especially motorcycles. 15

The clinical diagnosis group with the highest number of cases was peripheral neuropathies. The main diagnosis in the peripheral neuropathies group was CTS. This finding is consistent with the literature because CTS is considered the leading cause of focal peripheral compression neuropathy. 16 In the peripheral neuropathies group, despite the high frequency of idiopathic neuropathies, these rates can be found even in places with extensive access and resources for diagnostic investigation. 17

We added the osteoarticular disease group due to the large number of cases in which the evaluation did not reveal any neurological condition. Some cases represent degenerative spine diseases (cervical and lumbar spondylosis). Others correspond to idiopathic cavus foot with no alterations in the neurological examination and complementary tests, mainly electrophysiological. 18 19 In approximately two thirds of patients, clinical findings of cavus foot are associated with a primary neurological condition, most notably hereditary sensory-motor neuropathy, or Charcot-Marie-Tooth disease. Advanced cases of cavus foot, regardless of a neurological origin or not, may require orthopedic surgical correction to improve ambulation. 5 Some cases involve diagnostic uncertainty in evaluating idiopathic or non-neurological cavus foot, representing a diagnostic challenge. 20

Epilepsy represented the third group in the frequency of cases. It is among the most important neurological causes of long-term disability and increases the risk of fractures by two to six times compared to the general population. 21 22 Epileptic seizures lead to fractures, but they can also originate from cases of traumatic brain injury (TBI) associated with polytrauma. 21 23 Furthermore, primary epilepsy and the use of anticonvulsants since childhood can lead to cognitive deficits, with a subsequent impact on the educational level, resulting in jobs that require heavy lifting, with higher risks of orthopedic trauma. 24 Moreover, bone mineralization disorders and sedation due to antiepileptic drugs are risk factors for fractures. We found no literature on the issue despite the fear of orthopedic surgical failure due to postoperative epileptic seizures. This concern might be more justifiable in cases of refractory epilepsy or generalized tonic-clonic seizures. 22 25 Among the causes of developmental disorders, we found a high incidence of CP. With the national incidence estimated at 7 per 1,000 live births in Brazil, CP often generates secondary orthopedic conditions, such as neuromuscular scoliosis, hip dislocation, or equinus foot. 26 27

Our results demonstrate a high frequency of cervical spondylotic myelopathy (CSM) among neuromuscular diseases. Cervical spondylotic myelopathy corresponds to the main cause of myelopathy in the general population, usually in individuals over 50 years of age. The second most common diagnosis was neuromuscular scoliosis (NMS), followed by ALS. We must consider highlighting the differential diagnosis between CSM and ALS to avoid misdiagnosis and even overtreatment. 3

The high-frequency of NMS in the sample may be associated with the large number of CP cases found in the developmental disorders group. The differentiation between neuromuscular and idiopathic scoliosis is fundamental because of elevated failure rates with corrective orthopedic surgeries for the former. Neuromuscular scoliosis also involves higher bleeding rates and risk of infectious complications than idiopathic scoliosis surgery. 27

We found 10 HTLV-associated myelopathy **/** tropical spastic paraparesis (HAM/TSP) cases in the neuromuscular diseases group not demonstrated in the results. HTLV-associated myelopathy **/** tropical spastic paraparesis is one of the leading causes of myelopathy in developing countries as it is misdiagnosed as spinal cord compression by structural disease of the spine, thus causing unnecessary orthopedic surgeries. 28

Our results indicate a high frequency of psychiatric disorders. The neurological consultations at the unit are not open to the general population, decreasing access errors. These cases often consist of somatoform disorders, encompassing simulative, conversive, and somatization disorders. The large number of psychiatric patients may represent patient resistance to these conditions or the health professional's preference of referring them to a neurologist for fear of eroding the doctor-patient relationship by hypothesizing a psychiatric disorder. Moreover, patients may present lower rejection levels to a neurological evaluation than to a psychological or psychiatric one. 29

Considering the age distribution among patients with neurological diagnoses in our sample, the main group with underage patients was the developmental disorders. We do not find cases of the most common diagnosis, CTS, in underage patients. The presence of CTS in this population suggests the diagnosis of mucopolysaccharidosis or local compression by tumors. 30 We found no cases of NMS in the elderly. An unfavorable outcome of NMS from primary etiologies such as Duchenne dystrophy, spinal muscular atrophy, or cerebral palsy can explain these results. 27 Possibly for the same reason, we found no cases of intellectual disability in the elderly. However, the causes of intellectual disability are more extensive, with most cases being idiopathic. 31

Among the study's limitations is the fact that we could not evaluate the neurological care provided in the intensive care unit (ICU). Moreover, neurological referrals were provided on-demand by clinicians, and mainly orthopedists of the hospital and not due to spontaneous requests by patients. This fact may have affected the results, not representing the hospital's absolute frequency of neurological pathologies.

In conclusion, the knowledge of the profile of neurological disorders in a tertiary center of another medical specialty can improve the professional development of both specialties. Consequently, this interdisciplinary care can also optimize healthcare resources and reduce misdiagnosis, unnecessary treatments, and disabilities. We propose new studies of the profile of neurology referrals from tertiary hospitals guided toward other specialties to explore the diseases with the highest prevalence and incidence in various clinical practice scenarios.
